# Fibrinogen supplementation enhances antivenom efficacy in treating coagulopathy due to hemotoxic snakebite envenoming by *Trimeresurus* and *Agkistrodon* species in Yunnan, China

**DOI:** 10.3389/fmed.2025.1667800

**Published:** 2025-12-18

**Authors:** Qinfen Gao, Yi Wang, Yajun Teng, Qunyan Huang, Ya Wang, Shuai Feng, Bin Han, Zengzheng Li

**Affiliations:** 1 Department of Emergency, The First People's Hospital of Yunnan Province, Affiliated Hospital of Kunming University of Science and Technology, Kunming, China; 2 Department of Emergency, The First People's Hospital of Xuanwei City, Xuanwei, China; 3 Department of Emergency, The People's Hospital of Linxiang District, Lincang, China; 4 Department of Hematology, The First People's Hospital of Yunnan Province, Affiliated Hospital of Kunming University of Science and Technology, Kunming, China; 5 Yunnan Province Clinical Research Center for Hematologic Disease, The First People's Hospital of Yunnan Province, Kunming, China; 6 Yunnan Province Clinical Center for Hematologic Disease, The First People's Hospital of Yunnan Province, Kunming, China

**Keywords:** snake bite, fibrinogen, coagulation function, antivenom, hemotoxin

## Abstract

**Background:**

Timely administration of antivenom remains the cornerstone of treatment for hemotoxic snakebite envenoming, primarily aimed at neutralizing circulating toxins and halting the progression of venom-induced consumption coagulopathy (VICC), thus facilitating gradual recovery of the hemostatic system. However, immediate access to antivenom is not always possible, and variations in venom composition among snake species, along with individual patient differences, can result in significant morbidity (including persistent complications of coagulopathy) and mortality.

**Methods:**

This retrospective study evaluated 116 cases of hemotoxic snakebite envenoming caused by *Trimeresurus stejnegeri, T. mucrosquamatus*, and *Agkistrodon halys* at three hospitals in Yunnan Province, China (The First People's Hospital of Yunnan Province 63 cases, The People's Hospital of Linxiang District 28 cases, and The First People's Hospital of Xuanwei City 25 cases). Among these, thirty-three patients consented to receive adjunctive therapy with fibrinogen (Fg) in addition to standard antivenom treatment (*Agkistrodon acutus* antivenom or *Agkistrodon halys* antivenom), while the remaining 83 received antivenom alone. Coagulation parameters were measured at admission and discharge. Statistical analyses were performed using IBM SPSS Statistics and GraphPad Prism, employing the Mann-Whitney U test for non-normally distributed data and Student's *t*-test for normally distributed data.

**Results:**

In the antivenom-only group, significant reductions were observed in prothrombin time (PT), international normalized ratio (INR), activated partial thromboplastin time (APTT), and D-dimer (DD2) levels (all *P* < 0.05), accompanied by increases in Fg and fibrin degradation products (FDP; *P* < 0.05). Patients receiving the combined regimen demonstrated decreases in PT, INR, thrombin time (TT), APTT, FDP, and DD2 (all *P* < 0.05), along with a significant rise in Fg levels (*P* < 0.05). Those who received Fg presented with more severe coagulation deficits at baseline. Despite this, by the time of discharge, the median Fg level in the combination group [1.94 (1.52–2.20) g/L] was significantly higher than in the antivenom-only group [0.84 (0.68–1.17) g/L] (*P* < 0.0001), and closer to the physiological range (2–4 g/L). Moreover, the hospital stay in the combination group (4.88 ± 1.47 days) was significantly shorter than in the antivenom-only group (7.07 ± 2.02 days; *P* < 0.0001).

**Conclusions:**

These findings suggest that adjunctive Fg supplementation may improve the laboratory parameters of coagulopathy and reduce the length of hospital stay in hemotoxic snakebite envenoming. However, further clinical evaluations are needed to support the use of Fg as an adjuvant in the management of hemotoxic snakebite envenoming.

## Introduction

Only about 20% of the approximately 4,000 snake species worldwide are venomous (1, 2). Snakebite envenomation predominantly occurs in tropical regions near the equator. Each year, an estimated 1.8–2.7 million people are affected globally, leading to approximately 80,000–138,000 deaths and around 400,000 cases of long-term complications (1, 2). It primarily affects tropical regions on either side of the equator, with high incidences reported in Southeast Asia, sub-Saharan Africa, and South America (3). In China, it is estimated that over 100,000 snakebite incidents occur annually, with a mortality rate ranging from 5% to 10% and a disability rate between 25 and 30%. Most affected are individuals aged 50 years or older (1, 4). Venomous snakes are clinically classified into neurotoxic, hemotoxic, and cytotoxic snakes based on the toxic effects of snake venom on the human body. In China, Common venomous snakes are classified according to their main toxins: (1) neurotoxic snakes: *Bungarus multicinctus, Bungarus fasciatus, Ophiophagus hannah, Hydrophis caerulescens, Hydrophis fasciatus, Hydrophis cyanocinctus*; (2) hemotoxic snakes: *Trimeresurus stejnegeri, Deinagkistrodon acutus, Protobothrops mucrosquamatus, Rhabdophis subminiatus*, and *Rhabdophis tigrinus*; (3) cytotoxic snakes: *Naja atra* (33). In our previous epidemiological investigation, it was found that in Yunnan, snakes with hemotoxic venom are relatively common, mainly causing symptoms such as coagulopathy and local tissue damage. Common venomous snakes include the genera *Trimeresurus* (e.g., *T. stejnegeri, T. mucrosquamatus*) and *Agkistrodon* (e.g., *A. halys*) (5). Associated complications may include disseminated intravascular coagulation, defibrination syndrome, and various procoagulant coagulopathies (6, 7). The variation in coagulation cascade disturbances, even among bites from the same snake species, indicates that conventional coagulation pathways may not fully explain the underlying mechanisms of VICC (8). Prompt administration of correct antivenom is essential for neutralizing life-threatening venom toxins (8). Although some treatment centers provide blood transfusion therapies such as fresh frozen plasma and coagulation factors, existing research suggests these interventions may carry risks in cases of VICC, suggesting these intervention may worsen coagulation disturbances (9–11). However, there are studies which have shown that administering coagulation factors, cryoprecipitates, and fresh frozen plasma can help correct coagulation abnormalities in patients without negatively affecting clinical outcomes (12–14).

Blood transfusion therapy continues to be a subject of debate in the management of snakebite-induced coagulopathy (15–17). There remains a pressing need for alternative approaches to address coagulation dysfunction, as the most effective strategy for correcting these abnormalities is yet to be established. Evidence from several studies suggests that Fg concentrates may offer early and effective restoration of impaired fibrin polymerization, therefore reducing the risk of substantial blood loss (15–18). The regulatory effects of snake venom on the blood system can be classified into four categories: procoagulant components (including factor V activator, factor X activator, prothrombin activator and procoagulant enzyme) can accelerate the blood coagulation process; anticoagulant components (including factor IX/X binding protein, protein C activator, thrombin inhibitor and phospholipase A2) can inhibit coagulation function; fibrinolytic components (including plasmin and plasminogen activator) can dissolve the formed thrombus; and vascular injury components (including snake venom metalloproteinases, disintegrins and C-type lectins) directly damage the structure of the vascular wall (7, 19–22). Thrombin-like enzymes or fibrinogenases commonly cleave the α or β chains of Fg, generating fibrinopeptide A or B. This process depletes Fg without forming fibrin, ultimately impairing clot formation and causing severe spontaneous bleeding (19, 23). Fg, a key component of the hemostatic system, is typically present in healthy individuals at relatively high concentrations, ranging from 2 to 4 g/L (23, 24). During episodes of severe bleeding, Fg levels are often the first to decrease, reflecting its rapid consumption, dilution, and degradation through fibrinolysis (17). Fg is both safe and effective in treating bleeding associated with acquired hemorrhagic conditions and inherited Fg disorders, with a low incidence of adverse events (25–28). This positions it as a frontline therapy for controlling intraoperative bleeding related to hemostatic dysfunction (29).

This study evaluated the clinical outcomes of 116 patients with hemotoxic snakebite envenoming. Among them, 33 received a combination of antivenom and Fg therapy, while the remaining 83 were treated with antivenom alone. The objective was to determine whether adding Fg as an adjuvant in the management of hemotoxic snakebite envenoming could more effectively support the restoration of coagulation function compared to antivenom treatment alone.

## Materials and methods

### Inclusion and exclusion criteria

This retrospective study analyzed snakebite cases treated between June 2023 and October 2024 at The First People's Hospital of Yunnan Province (*n* = 63), The People's Hospital of Linxiang District (*n* = 28), and The First People's Hospital of Xuanwei City (*n* = 25). In total, 116 patients who showed signs and symptoms of hemotoxic snakebite envenoming were selected. Data collected included patient age, gender, snake species, and use of antivenom. Blood samples were collected at two time points (upon admission and within a 6 h period preceeding discharge) to assay coagulation parameters (lines 163−165 underlined in the manuscript). These parameters included prothrombin time (PT; reference range: 11.0–15.0 s), international normalized ratio (INR; 0.8–1.2), thrombin time (TT; 14.8–20.8 s), activated partial thromboplastin time (APTT; 28.0–43.5 s), fibrinogen (Fg; 2–4 g/L), fibrin degradation products (FDP; 0–5 μg/ml), and D-dimer (DD2; 0–0.5 μg/ml). All coagulation tests were conducted using the STA-R Evolution automated coagulation analyzer and compatible reagents (STAGO, France).

The use of antivenom was guided by the Chinese guidelines for management of snakebites (33), which relies on a combination of clinical presentation and available information to diagnose hemotoxic envenoming (33). The clinical signs of hemotoxic snakebite envenoming mainly include bleeding from the wound, skin petechiae, ecchymosis, gingival bleeding, hematemesis, melena, hemoptysis, and hematuria (5, 33). In severe cases, bleeding in important organs such as the brain may occur, as well as hypovolemic shock in severe cases (5, 33). Patients with comorbidities unrelated to snakebites, such as malignancies, immune disorders, pre-existing coagulation abnormalities, cardiovascular or cerebrovascular diseases, and pregnancy, were excluded from the analysis. The selection of antivenom used in the snakebite envenoming management was based on The Chinese guideline for management of snakebites. *Agkistrodon halys* bite uses antivenom against *Agkistrodon halys* envenomation (6,000 U per vial). *Trimeresurus stejnegeri* and *Trimeresurus mucrosquamatus* bites are treated with antivenom against *Deinagkistrodon acutus* envenomation (2,000 U per vial), as per the Chinese guideline (33) (Table 1). The two antivenoms products used in this study are produced by Shanghai Sialon Biotechnology Co., LTD. (Shanghai, China), with with the National Drug Approval number S10820180 with a 36 month validity. All the antivenoms used in the study were within their validity period and were uniformly purchased by the hospital and provided by the hospital pharmacy. In addition, as *Clostridium tetani* hidden in the snake's mouth poses a risk of causing tetanus, all patients were injected with tetanus immunoglobulin. According to the Visual Analog Scale (VAS) score >7, acetaminophen was given for pain relief treatment. When patients experience antivenom allergy (without shock) or still have swelling and pain after treatment with adequate antivenom, dexamethasone is given for symptomatic treatment. Patients with infection and tissue necrosis should use ceftriaxone (Table 1) (30).

**Table 1:** Information on the drugs used in the management of hemotoxic snakebite in the study area.

**Drug name**	**Dosage**	**Frequency**	**Indications**
Acetaminophen	0.5 g	Immediately, no more than 3 g per day, po	Pain
Dexamethasone	10 mg	Once a day, intravenous drip	Allergy to antivenom or persistent swelling and pain after treatment with adequate antivenom
Ceftriaxone	2 g	Once a day, intravenous drip	The wound gets infected or the tissue necrotizes
Tetanus immunoglobulin	250 UI	Immediately, im	All patients use it
*Agkistrodon acutus* antivenom	12,000 UI	Immediately, intravenous drip	*Agkistrodon halys* bite
*Agkistrodon halys* antivenom	8,000 UI	Immediately, intravenous drip	*Trimeresurus stejnegeri* and *Trimeresurus mucrosquamatus* bites

The study involving human participants was reviewed and approved by the Ethics Committee of the First People's Hospital of Yunnan Province (Approval No: KHLL2024-KY087), with a validity period from January 1, 2024, to December 31, 2025. The research was conducted in accordance with the local legislation and institutional requirements. Written informed consent for participation was obtained from all patients prior to the administration of fibrinogen therapy, in accordance with hospital policy and insurer requirements. For patients treated with antivenom alone, data were analyzed retrospectively under the waiver of consent granted by the ethics committee.

### Administration of Fg

This study administered treatment protocols across all three hospitals following the Chinese Guideline for the Management of Snakebites (30). As the current treatment for Fg is not recommended for snake bite patients, the use of Fg is adapted from the application of Fg in acute bleeding situations such as postpartum hemorrhage and cardiac surgery (26, 27, 31). There were thirty-three patients in the cohort who received Fg after administration of antivenom. The Fg supplementation was administered intravenously, with dosing determined according to the patient's measured Fg levels. The required dose was calculated using the formula:


$$\begin{array}{r} \left(2\text{ actual Fg level in g}/\text{L}\right)\;\times \text{ body weight }\left(\text{kg}\right)\;\times \;0.07\\ =\text{ required Fg dose }\left(\text{g}\right) \end{array}$$

In this formula, 2 g/L represents the target Fg concentration, and the factor 0.07 reflects the average plasma volume, constituting approximately 6%−8% of total body weight. Each patient received a single calculated dose based on their initial laboratory values; no subsequent dose adjustments were made based on follow-up testing. All patients receiving Fg supplementation were treated following this standardized dosing protocol. Fg (0.5 g/bottle) products were from Shanghai RAAS Blood Products Co., Ltd. and were to be stored in a light-proof environment at 2–8 °C. Before use, Fg (0.5 g/bottle) and sterilized water for injection (25 ml) were preheated to 30–37 °C. Sterilized water for injection was pre-heated by placing in a water bath at 30–37 °C and thereafter gently shaken to completely dissolve the product (vigorous shaking was avoided to prevent protein denaturation). Intravenous infusion was performed using an infusion set with a filter screening device. The drop rate was set at 60 drops per minute and Fg was used within 2 h after the administration of antivenom.

### Data analysis

The normality of the data was assessed using the Shapiro–Wilk test and Q–Q plots. The Mann–Whitney *U* test was used for data with non-normal distribution, and the Student's *t*-test was used for data with normal distribution. The Mann–Whitney *U* test was applied to compare coagulation parameters (PT, INR, TT, APTT, Fg, FDP, and DD2) between the two treatment groups at admission and discharge, as these data were non-normally distributed. In our data, only the length of hospital stay conformed to the normal distribution and was expressed as mean ± standard deviation. Continuous variables are presented as medians with interquartile ranges (IQR), while categorical variables are expressed as frequencies and percentages. Moreover, 95% confidence intervals (CIs) were reported for the median values within the IQR. Data visualization was performed using GraphPad Prism 7, and statistical analyses were conducted using IBM SPSS Statistics version 21.0 (Armonk, NY, USA). A two-sided *P*-value of < 0.05 was considered statistically significant. All graphical representations display medians along with their corresponding 95% confidence intervals.

## Results

### Basic characteristics of patients

This study analyzed 116 patients with hematotoxic snakebites, comprising 72 males and 44 females, with a median age of 55 years (range: 13–85 years). The bites were attributed to several snake species: *Trimeresurus stejnegeri* (*n* = 17), *Trimeresurus mucrosquamatus* (*n* = 81), *Agkistrodon halys* (*n* = 14), and unidentified species (*n* = 4). Among the cases we included, the median time from bite to treatment was 4 (3–6) hours, and all patients received no treatment before being sent to the hospital for treatment. The occupations of the included patients mainly included farming (65.52%, 76/116), workers (mainly engaged in forestry activities; 13.79%, 16/116), unemployed (20.69%, 14/116), and others (such as tourists and herdsmen, etc.; 8.62%, 10/116). Of the total cohort, 33 patients received a combination therapy of antivenom and Fg, including 19 cases of *Trimeresurus mucrosquamatus*, six of *Trimeresurus stejnegeri*, five of *Agkistrodon halys*, and three unidentified. The remaining patients (*n* = 83) were treated with antivenom alone. Beyond treatment differences, no additional matching or grouping criteria were applied. According to the Chinese Guideline for the Management of Snakebites (30), the clinical severity of our patients was moderate. A total of 153 g of Fg was used in this study, with an average of 4.64 ± 1.40 g of Fg used per patient. Whether it was the combined treatment group or the group treated only with antivenom, the average usage of antivenom per person was 3 ± 1 vials. Fg depletion (defined as Fg < 2 g/L) was observed in 70.69% of patients (82/116), with a median Fg level of 0.82 g/L (IQR: 0.60–1.23). Among the 82 patients with Fg depletion, 62.20% (51/82) showed wound bleeding. Patients (96.23%, 51/53) with bleeding had Fg depletion, with a median Fg level of 0.65 g/L (IQR: 0.60–1.07). In comparison, patients without bleeding symptoms (37.80%, 31/82) had significantly higher Fg levels, with a median of 1.32 g/L (IQR: 1.19–1.83; *P* = 0.005). These findings suggest a strong association between the severity of clinical symptoms, particularly bleeding, and the extent of Fg consumption. In addition, in our cohort, the average length of hospital stay for patients treated with antivenom combined with Fg (4.88 ± 1.47 days) was shorter than that of patients treated only with antivenom (7.07 ± 2.02 days; *P* < 0.0001; Table 1). Baseline characteristics of the study population are presented in Table 2.

**Table 2:** Sociodemographics, snake species, bite location, clinical presentation, and hospital stay of the study participants.

**Variable**	**With Fg (*n* = 33)**	**Without Fg (*n* = 83)**
Male/Female	17/16	55/28
Age (years)	55 (47.5–65)	50 (44–65)
**Species**		
*Trimeresurus stejnegeri* (*n* = 17)	6	11
*Trimeresurus mucrosquamatus* (*n* = 81)	19	62
*Agkistrodon halys* (*n* = 14)	5	9
Unknown (*n* = 4)	3	1
**Area bitten**		
Limbs bitten (*n* = 112)	96.55%	
Torso bites (*n* = 3)	2.59%	
Head and neck bites (*n* = 1)	0.86%	
**Prominent signs and symptoms**		
Local pain, bleeding wounds, local ecchymosis/petechiae (*n* = 20)	17.24%	
Local pain, local swelling, bleeding wounds (*n* = 26)	22.41%	
Bleeding wounds (*n* = 7)	6.03%	
Local pain, Local swelling (*n* = 26)	22.41%	
Local petechiae/ecchymosis (*n* = 14)	12.07%	
Local pain, Local swelling, Local Ecchymosis (*n* = 23)	19.83%	
Average length of hospital stay (Days)	4.88 ± 1.47	7.07 ± 2.02^***^
Average dose of Fg(g)	4.64 ± 1.40	Na
Dosage of antivenom(bottle)	3 ± 1	3 ± 1

“^***^”: Indicates P < 0.001, with a statistically significant difference; Student's T-test were used for comparison. “ns”: Indicates no statistical difference, and the Mann-Whitney U test was used for comparison.

### Coagulation indicators of the patient upon admission

We initially compared the pre-treatment coagulation baseline parameters of patients who received antivenom combined with Fg supplementation upon admission with those who only received antivenom serum. The results showed that PT/s, APTT/s, FDP, and D-dimer levels of the Fg-treated group were significantly higher than those of the non-Fg group (*P* < 0.05; Table 3). However, Fg levels before treatment in the Fg-treated group was significantly lower than that of the non-Fg group (*P* < 0.05; Table 3). Therefore, patients with more severe coagulation dysfunction upon admission received fibrinogen supplementation.

**Table 3:** The differences in coagulation indicators at admission between patients treated with antivenom combined with Fg and those treated with antivenom alone.

**Parameters**	**Without Fg (Median, IQR)**	**95%CI for Median**	**With Fg (Median, IQR)**	**95%CI for Median**	***P* value**
PT/s	12.40 (11.50–14.20)	11.90–14.10	15.10 (13.40–20.45)	13.8–17.7	0.0005
INR	1.07 (0.99–1.22)	1.04–1.23	1.10 (1.03–1.44)	1.04–1.34	0.7886
TT/s	22.15 (20.13–25.68)	21.05–24.90	25.10 (20.85–34.90)	22.45–32.80	0.2351
APTT/s	23.55 (19.87–27.02)	22.50–26.30	34.90 (31.80–42.40)	32.25–38.9	< 0.0001
Fg g/L	0.82 (0.60–1.59)	0.54–1.05	0.78 (0.55–1.16)	0.60–1.23	0.0034
FDP μg/ml	0.90 (0.40–2.57)	0.47–1.19	46.77 (21.70–86.97)	31.23–63.80	< 0.0001
DD2 μg/ml	1.52 (0.98–2.22)	1.58–2.41	17.37 (3.62–45.00)	5.64–40.96	< 0.0001

P < 0.05: there is a statistical difference, P > 0.05: there was no statistical difference.

### Coagulation indicators of patients upon discharge

**Figure 1:**
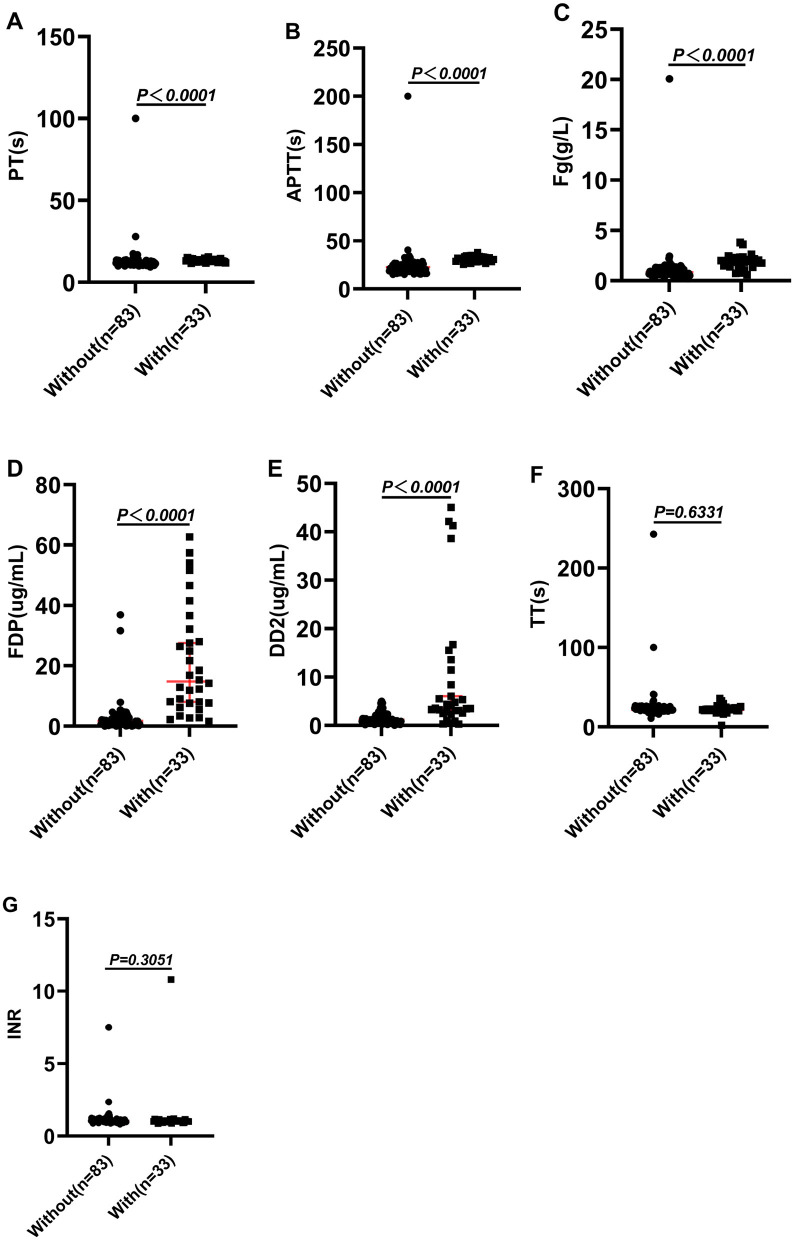
The differences in coagulation indicators at discharge between patients treated with antivenom combined with Fg and those treated with antivenom alone. **(A)** PT (s), **(B)** APTT (s), **(C)** Fg (g/L), **(D)** FDP (μg/ml), **(E)** DD2 (μg/ml), **(F)** TT (s), **(G)** INR. *P* < 0.05: there is a statistical difference, *P* > 0.05: there was no statistical difference.

**Figure 2:**
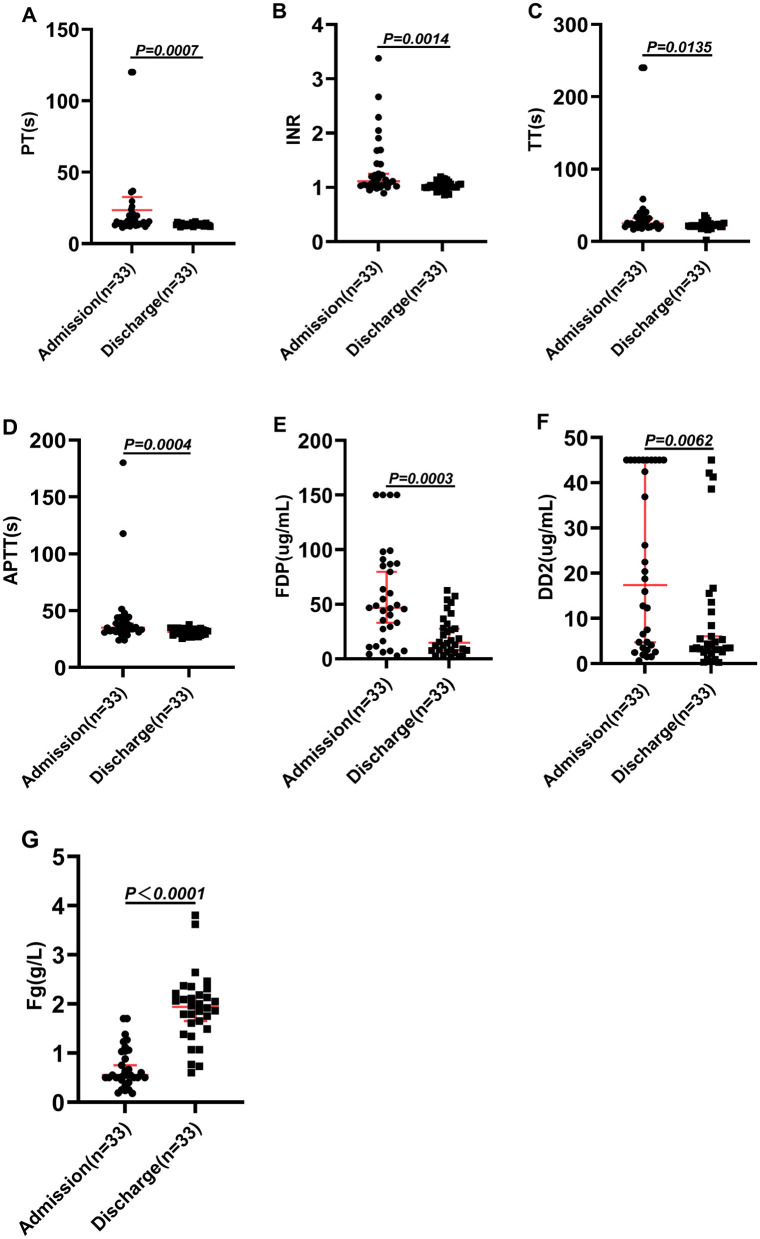
Comparison of coagulation indicators at admission and discharge in patients treated with antivenom combined with Fg. **(A)** PT (s), **(B)** INR, **(C)** TT (s), **(D)** APTT (s), **(E)** FDP (μg/ml), **(F)** DD2 (μg/ml), **(G)** Fg (g/L). *P* < 0.05: there is a statistical difference, *P* > 0.05: there was no statistical difference.

As shown in Table 3, patients who received antivenom in combination with Fg presented with more pronounced coagulation dysfunction at admission. We then analyzed coagulation parameters at discharge between the two groups. The findings revealed that although PT (reference range: 11.0–15.0 s) and APTT (reference range: 28.0–43.5 s) remained slightly higher in the Fg-treated group compared to the non-Fg group, both values fell within normal reference limits (Table 4 and Figure 1). Furthermore, levels of Fg, FDP, and DD2 were significantly higher in the combination therapy group (*P* < 0.05; Table 4 and Figure 1). The Fg level is particularly interesting, as it is a critical marker of coagulation status. Fg, the most abundant coagulation factor in circulation, typically ranges between 2–4 g/L in healthy individuals (23). Following combination therapy, the hospital stay of the patients was significantly shortened (Table 2), and the Fg concentration at discharge reached 1.94 (1.52–2.20)g/L, which approached the normal physiological range and were significantly higher than those observed in patients treated with antivenom alone, whose Fg levels were 0.84 (0.68–1.17) g/L (Table 4, Figures 1, 2). the length of hospital stay for patients in the combined group was significantly shorter (4.88 ± 1.47 days) than for patients in the antivenom only group (7.07 ± 2.02 days; Table 2). This improvement may reflect improved recovery of coagulation function in cases of coagulopathy resulting from snake envenomation. Figure 2 illustrates the changes in coagulation parameters between admission and discharge among patients who received antivenom in combination with Fg.

**Table 4:** The differences in coagulation indicators at discharge between patients treated with antivenom combined with Fg and those treated with antivenom alone.

**Parameters**	**Without Fg (Median, IQR)**	**95%CI for Median**	**With Fg (Median, IQR)**	**95%CI for Median**	***P* value**
PT/s	13.30 (12.60–14.05)	12.60–13.60	12.10 (11.2–13.10)	11.20–12.50	< 0.0001
INR	1.01 (0.99–1.09)	0.99–1.04	1.05 (0.96–1.13)	0.97–1.09	0.3051
TT/s	21.60 (20.00–23.95)	20.15–22.40	21.60 (19.90–23.90)	20.20–23.20	0.6331
APTT/s	31.30 (28.90–33.45)	28.90–32.65	22.74 (19.50–25.50)	20.30–25.19	< 0.0001
Fg g/L	1.94 (1.52–2.20)	1.72–2.13	0.84 (0.68–1.17)	0.77–1.14	< 0.0001
FDP μg/ml	14.80 (7.62–31.11)	7.85–24.06	1.79 (0.68–2.94)	0.65–2.44	< 0.0001
DD2 μg/ml	3.50 (2.55–11.44)	2.84–5.48	1.18 (0.73–2.07)	0.98–1.36	< 0.0001

P < 0.05: there is a statistical difference, P > 0.05: there was no statistical difference.

## Discussion

Our study aimed to evaluate whether the addition of fibrinogen (Fg) to standard antivenom therapy could more effectively support the recovery of coagulation function in patients with hemotoxic snakebite envenoming. Our findings demonstrate that antivenom administration yields favorable outcomes in patients, aligning with the conclusions of most studies, which report that antivenom therapy is effective in managing coagulopathy resulting from hemotoxic snakebite envenoming (32–37). This finding suggests that Fg supplementation may have facilitated more effective restoration of coagulation function. However, this study mainly evaluated laboratory indicators. Due to the lack of complete detection of coagulation factors and the recovery time of coagulation function, the true recovery time of coagulation function and whether there will be residual coagulation dysfunction remain unclear. Therefore, further research is needed to clarify the clinical significance.

In treating snake bites, the primary goal of antivenom therapy is to neutralize the toxin rather than treat VICC (8, 38, 39). In our study, the combined use of antivenom and Fg therapy was primarily intended to support the recovery of coagulopathy while simultaneously neutralizing the venom. Our findings suggest that this combined approach significantly improves laboratory coagulation parameters and hospital stay (Table 4, Figures 1, 2), indicating a potential benefit for restoring hemostatic function. After hemotoxic snakebite envenoming, in addition to adequate antivenom and fibrinogen, individualized diagnosis and treatment plans should also be formulated based on the different clinical manifestations of each patient. This includes: allergy to antivenom, or persistent swelling and pain after treatment with adequate antivenom (30, 40). Acetaminophen can be used for pain relief (30). When there is a clear infection focus or tissue necrosis, amoxicillin/clavulanic acid, fluoroquinolones, cefazolin, third-generation cephalosporins or aminoglycosides are selected for the treatment of infection (41, 42). In addition, since *Clostridium tetani* may be carried in the mouth of snakes, tetanus human immunoglobulin should be routinely used to prevent tetanus (43, 44). In Table 1, we also expounded on the use of relevant drugs. It should be noted that some studies have shown that Traditional Chinese medicine treatment methods such as acupuncture and prescriptions may have the potential to improve local and systemic poisoning symptoms in patients (4, 30). However, Herbal decoction and herbal remedies are not recommended by WHO guideline (45).

Various countries and regions are actively exploring diverse treatment strategies, including fresh frozen plasma and blood transfusions, which have shown potential therapeutic benefits in certain cases (9–11, 46). However, these approaches also carry potential risks, including pathogenic contamination and immune-mediated reactions (18). As mentioned earlier, Fg is currently mainly applied in cases of acute hemorrhage (such as postpartum hemorrhage and cardiac surgery) (26, 27). In the early stages of low fibrinogen, rapid supplementation of Fg can promote the recovery of coagulation dysfunction in patients and reduce the risk of bleeding and death (47–49). In our study, The primary rationale for Fg supplementation is to increase Fg availability during antivenom-mediated neutralization of venom toxins, promoting fibrin formation and supporting the recovery of coagulation function. Despite this, DD2 levels remained significantly elevated in the combination therapy group compared to the antivenom-only group. This is likely attributable to the greater severity of coagulopathy among patients receiving Fg therapy at baseline (Table 3). In severe coagulopathy, Fg is converted into fibrin under the action of thrombin. Fibrin can further form fibrin monomers, which are cross-linked by activating factor XIII and then degraded by fibrinolytic enzymes to produce DD2 (19, 50). At the same time, fibrin can be decomposed by plasmin to produce FDP (51, 52). Moreover, the additional Fg administered to patients is also subject to degradation by fibrinolytic enzymes, contributing further to elevated DD2 levels (53). Therefore, patients receiving combined Fg therapy show higher DD2 and FDP levels at both admission and discharge. It is important to note that, in general, DD2 elevation resulting from coagulopathy does not typically lead to microthrombus formation (7, 37). However, during and after the supplementary treatment of Fg, it should be noted that there is a risk of thromboembolism in the supplementation of Fg (25–28). Therefore, monitoring coagulation function during treatment is essential to maintain Fg levels within the optimal 2–4 g/L range.

Overall, our findings suggest that combining antivenom with Fg supplementation effectively improves laboratory indicators of coagulopathy following snakebite and shorten the length of hospital stay, particularly in restoring Fg levels. This adjunctive therapy shows potential as a treatment strategy. However, our retrospective study has several limitations: (1) the presence of selection bias due to the lack of randomization or group matching; (2) the data were drawn from a single geographic region, limiting generalizability; (3) no long-term follow-up was conducted to assess adverse outcomes such as thrombosis or allergic reactions to Fg; (4) patients in the Fg group had significantly worse baseline coagulation profiles, introducing indication bias that may confound interpretation of the treatment effect; and (5) the lack of dynamic monitoring of coagulation function leads to the inability to determine the accurate time for the recovery of coagulation dysfunction. Therefore, further validation through larger, multi-center, and multi-regional prospective studies is essential. To address these limitations, we have established the “Yunnan Province Snakebite Research Collaboration Group,” our team remains committed to advancing research in this field.

## Data Availability

The original contributions presented in the study are included in the article/supplementary material, further inquiries can be directed to the corresponding authors.
